# Does a GP service package matter in addressing the absence of health management by the occupational population? A modelling study

**DOI:** 10.1186/s12913-024-10954-9

**Published:** 2024-05-17

**Authors:** Jing Guo, Ying Qian, Chen Chen, Hong Liang, Jiaoling Huang

**Affiliations:** 1 School of Social Development and Public Policy of Fudan University, Shanghai, China; 2 Business School, University of Shanghai for Science and Technology, Shanghai, China; 3 Pengpuxincun Community Health Service Center, Shanghai, China; 4 School of Public Health, Shanghai Jiao Tong University School of Medicine, Shanghai, China

**Keywords:** Primary healthcare, GP service package, Occupational population, Contract rate, System dynamics model

## Abstract

**Objective:**

To assess the influence of supply and demand factors on the contract behavior of occupational populations with general practitioner (GP) teams.

**Methods:**

We employed a system dynamics approach to assess and predict the effect of the general practitioner service package (GPSP) and complementary incentive policies on the contract rate for 2015–2030. First, the GPSP is designed to address the unique needs of occupational populations, enhancing the attractiveness of GP contracting services, including three personalized service contents tailored to demand-side considerations: work-related disease prevention (WDP), health education & counseling (HEC), and health-care service (HCS). Second, the complementary incentive policies on the supply-side included income incentives (II), job title promotion (JTP), and education & training (ET). Considering the team collaboration, the income distribution ratio (IDR) was also incorporated into supply-side factors.

**Findings:**

The contract rate is predicted to increase to 57.8% by 2030 after the GPSP intervention, representing a 15.4% increase on the non-intervention scenario. WDP and HEC have a slightly higher (by 2%) impact on the contract rate than that from HCS. Regarding the supply-side policies, II have a more significant impact on the contract rate than JTP and ET by 3–5%. The maximum predicted contract rate of 75.2% is expected by 2030 when the IDR is 0.5, i.e., the GP receives 50% of the contract income and other members share 50%.

**Conclusion:**

The GP service package favorably increased the contract rate among occupational population, particularly after integrating the incentive policies. Specifically, for a given demand level, the targeted content of the package enhanced the attractiveness of contract services. On the supply side, the incentive policies boost GPs’ motivation, and the income distribution motivated other team members.

**Supplementary Information:**

The online version contains supplementary material available at 10.1186/s12913-024-10954-9.

## Introduction

The global public health system has historically prioritized the health of older adults and children, but neglected those of the young and middle-aged. However, the occupational population is emerging as high-risk groups for diseases, including cardiovascular, musculoskeletal, (e.g., neck, shoulder, and back pain) [1, 2], and work-related (mental health, stress, and fatigue) diseases [3]. These health problems adversely affect employee productivity, business costs, and national health-care expenditures [4]. As primary care resources focus on improving health in socially disadvantaged populations, the suboptimal health status of the occupational population is challenging to identify and is frequently overlooked [5]. Nonetheless, the escalation of their suboptimal health status increases disease susceptibility and serves as a crucial warning signal to engage in health management and promotion [6]. 

Primary health care is considered as a whole-of-society approach to health [7], and its role in health management and promotion has been widely substantiated [8]. Integrating work-related health issues into primary health care facilitates the timely detection and intervention in occupational health concerns, effectively mitigating prolonged sick leave and work incapacity [9]. Furthermore, studies have widely acknowledged that the extension of primary health care into occupational health through the provision of basic occupational health services yields economic benefits for businesses and job satisfaction for the occupational population [10, 11]. Research has demonstrated that well-planned occupational safety and health measures can generate economic returns that exceed the initial monetary investment by 3–10 times [12], and that effective health promotion programs can reduce sickness absenteeism by 27%, health-care costs by 26%, and insurance costs by 32% [13]. In regions with well-established primary health-care systems, such as the United Kingdom and the Netherlands, primary health-care services have been expanded to include health services for occupational populations [14, 15]. Nevertheless, many countries continue to encounter challenges in establishing comprehensive occupational health services and are constrained by developing primary health-care systems [16]. 

In China, the general practitioner (GP), also known as family doctors, serve as the gatekeeper of residents’ health by managing their overall well-being, addressing common illnesses, and controlling healthcare costs [17, 18]. Due to unequal healthcare development, residents often prefer higher-level hospitals, leading to concentrated resources and challenges such as the difficulty of getting medical service and high costs [19]. China’s general practitioner policy, introduced after the 2009 healthcare reform, is an important initiative to enhance the quality of primary healthcare and promote hierarchical diagnosis and treatment [20]. Notably, the promotion of GP contract services plays a crucial role in implementing the hierarchical diagnosis and treatment system [21]. This approach was reinforced by the issuance of the “Guiding Opinions on Promoting the Contracted Services of Family Doctors” in 2016. The policy strategically employs intrinsic motivation and external support mechanisms to augment public engagement in contracted services and encourage general practitioners to actively participate in these services [22]. China has developed diverse and distinctive contracting service models, with the “basic package + personalized package” model proving highly efficacious in elevating the health status of patients with chronic diseases [23]..

In 2019, China launched the “Healthy Enterprise” action plan to improve occupational health [24]. Nevertheless, health interventions for occupational populations by (GP) teams are far from sufficient, and this inadequacy poses significant challenges. Shanghai has always been a leader in China’s GP policy; in 2021, it issued a guideline to promote population health throughout the lifespan. The guideline encourages the expansion of community health services to functional communities (including enterprises, industrial parks, and commercial buildings) and encourages contracting with a GP team to establish healthy enterprises [25]. However, the 2023 data reveal a contract rate of around 37% among Shanghai’s resident population, with more than 90% being older individuals, and a low contract rate among young and middle-aged people [26]. There are two primary reasons for this low contract rate. First, from the demand perspective, most GP service packages are designed for older people, children, and individuals with chronic diseases [27], with no package designed specifically for occupational groups, resulting in a low GP contract rate within this demographic [28–30]. Second, on the supply side, GP teams confront a burdensome workload and substantial work-related pressures, without an effective incentive mechanism to enhance their work motivation [31, 32]. 

This study developed a system dynamics model to simulate contract behavior for occupational populations in response to a GP service package and complementary incentive policies. The package was designed to provide demand-side motivations by improving the attractiveness of the package, and supply-side motivations by improving incentives. The objective of this research is to examine the impact of the demand- and supply-side factors on the contract rate of the occupational population with GP teams. By simulating the tailored interventions on the contract rate, this study provides an evidence-based decision-making strategy to improve the health management practice among the occupational population. This paper is organized as follows: The Section 2 constitutes a literature review. Section 3 outlines the investigation methods, including study design, model structure, factors of the GP service package and complementary incentive policies, model formulation, and model validation. Section 4 presents the results of applying the proposed model in different scenarios to analyze the effects of the GP service package and complementary incentive policies on contract behavior. Section 5 discusses various influencing factors and concludes the paper.

## Literature review

System dynamics (SD) model is a frequently utilized approach in operations research, a discipline that employs quantitative or qualitative models to tackle intricate problems and facilitate decision-making [33]. SD has been widely applied in health care delivery, population health & health economics, substance abuse, infectious disease, biology & microbiology, health care products, mental health and health care education [34]. Some studies primarily focus on supply-side interventions, particularly concerning hospital capacity, addressing key factors such as bed utilization, nursing processes, admission policies, and the number or skills of medical personnel [35–38]. Some researchers concentrate on demand-side interventions, targeting various population subsets, including strategies for chronic disease management such as diabetes, heart failure, cardiovascular risk and disease [39, 40]. The application of system dynamics in health system reform is also considered capable of achieving balance between the supply and demand of healthcare services [41, 42]. Other widely used technical approaches include Agent-Based Modelling and Simulation (ABMS), Discrete Event Model (DEM), Monte Carlo Simulation, and so on [43, 44]. In recent years, particularly influenced by the Covid-19 epidemic, a substantial body of literature in operations research has emerged [45, 46], covering diverse topics such as infection-to-death prediction, COVID-19 testing, isolation strategies, vaccination strategies, and the effectiveness of regulatory interventions [47–50]. 

A number of studies has focus on clinical processes, demonstrating interest in aspects related to patients, diseases, or drugs [51–53]. For instance, they employ agent-based modeling and simulation (ABMS) to improve vascular access creation among hemodialysis patients [54], address the care delivery for patients with thrombotic and bleeding disorders [55], and explore its application in health care decision support [56]. Other researchers utilized the discrete event simulation (DES) to reduce on patient wait times and crowding [57]. For example, McKinley et al. [58] developed a DES model to evaluate the impact on system flow of a quality improvement (QI) initiative, specifically focusing on a time-specific protocol to reduce the time to antibiotic delivery for children with cancer and central venous catheters presenting to a pediatric emergency department with fever. Additionally, some researchers concentrate on healthcare costs [59], such as Silverman et al. [60] finding the three-level approach useful for helping health administrators navigate system complexities to find effective interventions at lower costs. Kunc and Kazakov [61] monitored the status of patients with chronic heart disease and its impact on the cost of the entire healthcare system (e.g., hospitals, patients, government) using a SD model, which provided the full range of the strategic planning process in healthcare.

Some researchers interested in the health care personnel resources. A variety of operations research methods are employed in studying nurse staffing, addressing issues such as the nurse rostering problem [62, 63] and nurse education [64]. Koichubekov et al. [65] predicted the primary health care personnel resource using a SD model, considering the flow of medical workers, demographic data of the population and the prevalence of the disease over time. The simulation results suggest an exacerbation of the shortage of primary care physicians, emphasizing the necessity for government measures to incentivize and support young medical doctors to become GPs. Similarly, Basu and Gupta [66] developed a SD model capable of simulating supply-side policy changes (e.g., increased international medical graduates, delayed retirements) and reflecting changes in demand (e.g., a cure for leukemia, varying work intensities for physicians). The model predicts the number of GPs, medical specialists, and surgical specialists, considering both supply and future demand. Similarly, Konneh et al. [67] employed agent-based modeling and simulation (ABMS) to study the influence of education, training, and performance of healthcare workers, revealing effective training and adherence to proper care procedures play a crucial role in preventing infections. Some scholars are also concerned about the effects of general practitioner policies. For example, Jiaoling Huang et al. [68] constructed a system dynamics model to examine whether the GP policy contributed to eliminating health disparities. The results showed that the GP policy would play a positive role in reducing health disparities in the initial stage, and medical price control rather than health management was the dominant mechanism.

Current literature highlights the significant potential of operations research methodologies, including models and simulations, in addressing healthcare challenges and decision-making [69]. Nevertheless, existing literature on promoting general practitioner contracting services frequently relies on static models, overlooking the dynamic and complex nature of policy systems [70–72]. These studies predominantly highlight the contracting by community or rural residents, overlooking the distinct characteristics of young and middle-aged occupational population. The pathways to promote the contracting of GPs by occupational populations are not yet clear. Unlike other studies that focus on either the supply or demand side, this research aims to fill this gap by addressing the pathways for contracting with GPs among occupational populations.

## Methods

### Study design

Based on the classical Bass diffusion model, we developed a system dynamics model in Vensim® 8.01 software to simulate the intervention effect of the GP service package (GPSP) and complementary incentive policies on GP contract behavior for the occupational population from both the demand and supply sides for 2015–2030. Additionally, key variables such as attractiveness, driving force, collaborative distribution et al., were identified as primary factors for intervention on supply and demand sides. Attractiveness in psychology denotes a positive assessment or preference for certain attributes of an object, or a combination of the relative importance of a personal interest and the perceived capacity of the object to fulfill individual benefits [73, 74]. Logically, attractiveness specifically refers to the appeal of GPSP among occupational population in this study. The higher the belief among occupational population that the GPSP can fulfill their health needs, the more attractive it becomes, increasing the likelihood of its selection. The “driving force” is commonly used to describe the motivation that induces change or behavior, including driving factors such as internal motivations, profit, achievement, and social values [75]. The driving force in this model primarily represents the factors that motivate GPs to enhance service quality, including Job Title Promotion (JTP), Income Incentive (II), Education & Training (ET), and collaborative distribution. The collaborative distribution specifically involves collaboration within the GP team, where GPs work in coordination with community nurses, public health physicians, rehabilitation therapists, and others to deliver high-quality services [21], operationalized in this study through Income Distribution Ratio (IDR), which allocates income based on the services provided and costs incurred by members of the GP team. Word-of-mouth is the communication between non-commercial communicators and receivers regarding brands, products, or services, considered a powerful force in shaping consumer behavior, with customer trust and satisfaction significantly influencing word-of-mouth communication [76]. In this study, the intervening factors for promoting the adoption from word-of-mouth primarily stem from improvements in service quality on the supply side and the increased attractiveness of GPSP on the demand side. To ensure the accuracy and objectivity of the model, two surveys were conducted concerning: (1) occupational population needs from the demand side; and (2) GP incentives from the supply side.

### Model structure

**Fig. 1:**
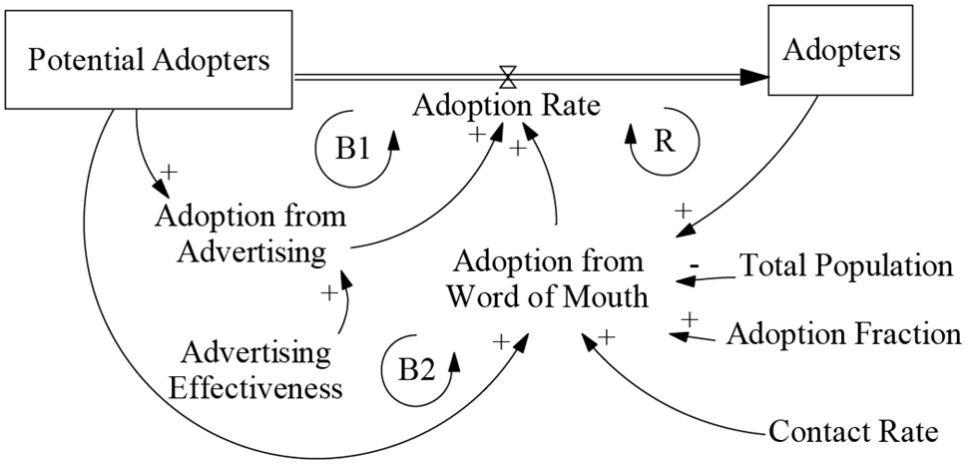
The Bass diffusion model

The contract rate examined in this study refers to the behavior of occupational populations contracting with GPs, which can be seen as a form of adoption, making it suitable for the Bass model. The original Bass model’s adoption rate is influenced by both word-of-mouth and advertising. Specifically, adoption driven by word-of-mouth is a reinforced loop influenced by adopters, potential adopters, the adoption fraction, the contact rate, and the total population to increase the attractiveness of adoption. Advertising is a balanced loop influenced by potential adopters and advertising effectiveness (Fig. 1) [77, 78]. 

Similarly, the SD model in this paper is a diffusion model primarily focused on examining the contract rate of young and middle-aged working individual. As such, the model comprises two main groups: occupational individuals and GPs. Occupational population is mainly derived from the young and middle-aged population aged 18–59 in Shanghai. GPs includes medical staff in community health service centers, secondary and tertiary hospitals. In addition, it is noteworthy that the GP is inherently part of the organization (community health service center) and can effectively represent the organization, eliminating the need to introduce an additional organization into the model. Potential contractors, akin to potential adopters in the Bass model, encompasses the young and middle-aged population who have not contracted with a GP. Contractors, akin to adopters in the Bass model, encompass occupational populations who contracted with a GP. Additionally, given that the contract is free of charge, residents have the flexibility to change GPs if dissatisfied with the contracted service, and there are few statistics on withdrawal individuals, the model does not incorporate individual withdrawals.

**Fig. 2:**
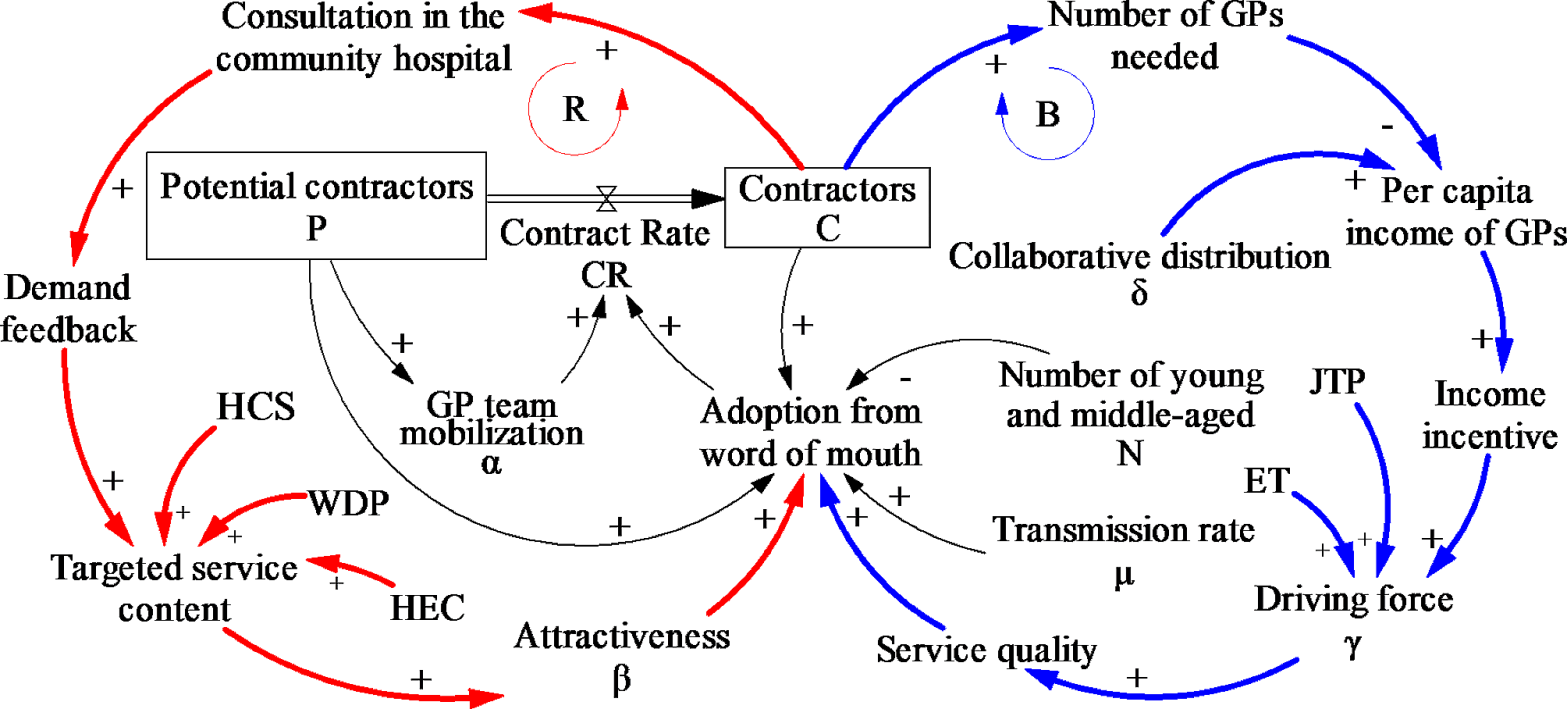
The extended model

The adoption of GP contract services, i.e., the contract rate, is molded by a combination of GP mobilization and word-of-mouth. GP team mobilization represents the effectiveness of GP teams in promoting contract services to patients during consultations or directly advertising contract services in the building. To further enhance word-of-mouth effects, the model is extended in two aspects: (1) on the demand side (red loop), the focus is on increasing the targeted service content to enhance the attractiveness of contract services to occupational groups, thereby increasing adoption based on word-of-mouth. The reinforcing loop primarily strengthens through the GPSP (HCS, WDP, HEC), which bolster the positive effects of service content to enhance the word-of-mouth effects of contracting. (2) On the supply side (blue loop), there exists a balancing loop, whereby the increase of GPs needed leads to a reduction in the per capita income of GPs, ultimately resulting in a negative impact on the contract rate. To counteract this adverse effect, the complementary incentive interventions (JTP, II, ET) and collaborative distribution, aimed at mitigating the negative impact of reduced income and enhancing the service motivation of GPs to facilitate contract rate. as illustrated in Fig. 2.

### Factors of GP service package and complementary incentive policies

The research team used questionnaires to analyze the personalized needs of 2,272 young and middle-aged members of occupational populations in Shanghai for GP contract services (See Appendix 1 of the Supplementary for detailed questionnaire and Appendix 3 for detailed factor analysis). Factor analysis was conducted using the principal component method, yielding three demand-side factors: health-care services (HCS), work-related disease prevention (WDP), and health education & counseling (HEC). Similarly, a factor analysis was conducted on 2,000 front-line medical staff in Shanghai (See Appendix 2 of the Supplementary for detailed questionnaire and Appendix 3 for detailed factor analysis), which identified three primary supply-side driving forces: income incentives (II), job title promotion (JTP), and education & training (ET). Given the collaborative nature of contract services, income distribution ratio (IDR) was incorporated as a factor on the supply side in the model. The main intervention variables of GPSP and complementary incentive policies on both demand and supply sides are shown in Table 1 and additional information is provided in Supplementary Table 11 of the Supplementary Appendix 4.

**Table 1:** Main variable settings

Main Variables	Formulas	Substitution
$\beta$: Attractiveness	${\upbeta }=$HCS impact + WDP impact + HEC impact	HCS (Health Care Service) without HCS= “0”, with HCS= “1”
		WDP (Work-related Diseases Prevention) without WDP= “0”, with WDP= “1”
		HEC (Health Education &Consultation) without HEC= “0”, with HEC= “1”
$\gamma$: Driving force	$\gamma =$minimum Driving force * (ET impact + JTP impact + II impact)	ET (Education & Training) without ET= “0”, with ET= “1”
		JTP (Job Title Promotion) without JTP= “0”, with JTP= “1”
		II (Income Incentive) with Lookup (per capita income of GPs)
$\delta:$Collaborative distribution	Policy Test	$\delta =$ IDR (income distribution ratio) = 0.1, 0.3, 0.5,0.7 or 0.9

### Model formulation

The GP contract diffusion process, examined in this study through the contract rate (CR), reflects the behavior of occupational populations contracting with GPs—a form of adoption well-suited for the Bass model. The CR is influenced by GP mobilization and word-of-mouth effects from both the supply and demand sides. In the extended model, we have:

1 $$ {{\text{Adoption from GP team mobilization}}={\alpha} *{\text{P}}}, $$

where the parameter $\alpha$, GP team mobilization, is the effect of GP mobilization on potential contractors during the service process; and P represents the number of potential contractors.

2 $$ {{\text{Adoption from word-of-mouth}}=\beta *\delta *\gamma *\mu *\text{P}*\text{C}/\text{N}}, $$

where $\beta$ represents the attractiveness effect, which is related to WDP, HCS, and HEC in the contract services; $\gamma$ represents the driving force, indicating the effects of II, JTP, and ET on the contract rate; $\delta$ represents the collaborative distribution effect, which is related to income distribution; $\mu$ represents the contract rate and indicates the spillover effect from contractors to potential contractors; C represents the number of contractors; and N denotes the population of young and middle-aged persons.

The GP contract diffusion model for occupational groups can be expressed as:

3 $$ {{\text {CR}}={\alpha *{\text{P}}+{\beta} *{\delta} *{\gamma} *{\mu} *{\text{P}}*{\text{C}}/{\text{N}}}}. $$

The main parameter settings and sources in the model are shown in Supplementary Table 12 of Supplementary Appendix 4.

### Model validation

**Fig. 3:**
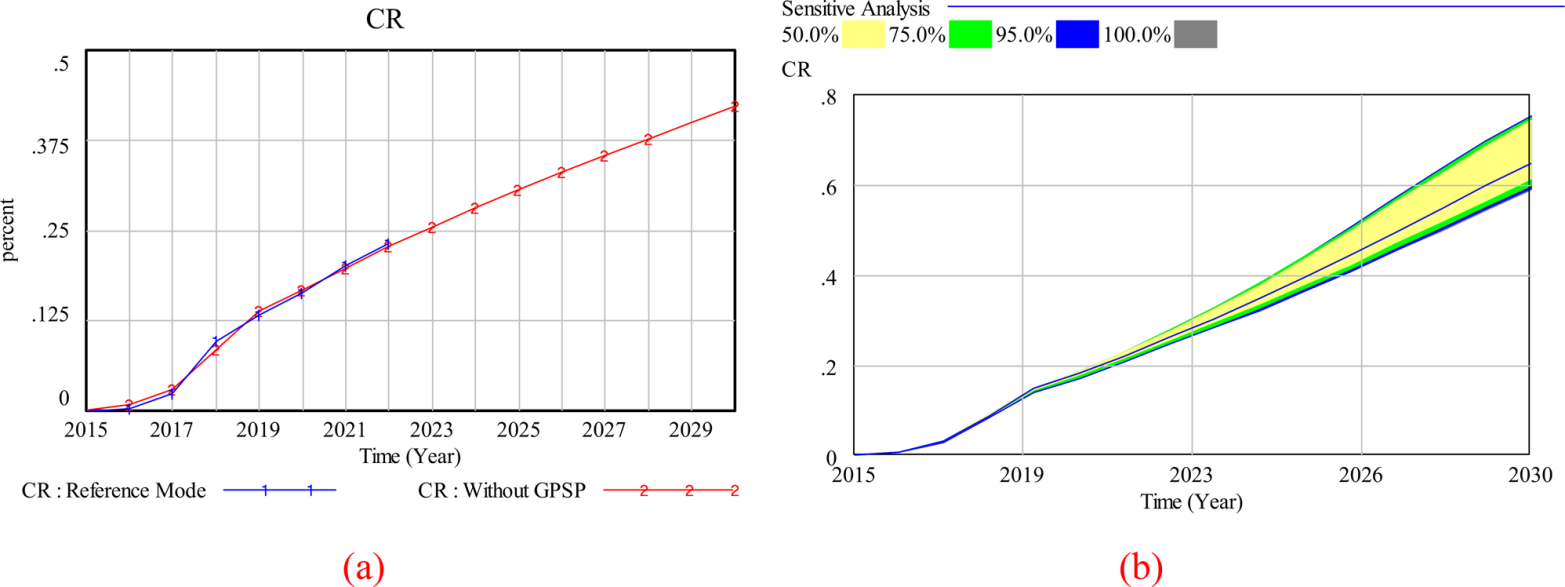
(**a**) historicity testing and (**b**) sensitivity analysis

The model development involves community health service center directors, medical department staff, GP teams, and relevant staff of community health service management centers to validate the key concepts, variables and model structure. The historical contract rate statistics, sourced from authoritative government offices, exhibit a high level of accuracy and credibility. As shown in Fig. 3(a), the simulated result is in good agreement with historical statistics data, which validated the effectiveness of the SD model. In addition, sensitivity analysis is also commonly seen in testing the accuracy of certain parameter estimates or system structures in the model. In this study, we conducted sensitivity analysis for income distribution ratio (Fig. 3(b)), which not only emphasizes its substantive importance but also greatly enhances confidence in the validity of the model.

## Results

### GP service package

**Fig. 4:**
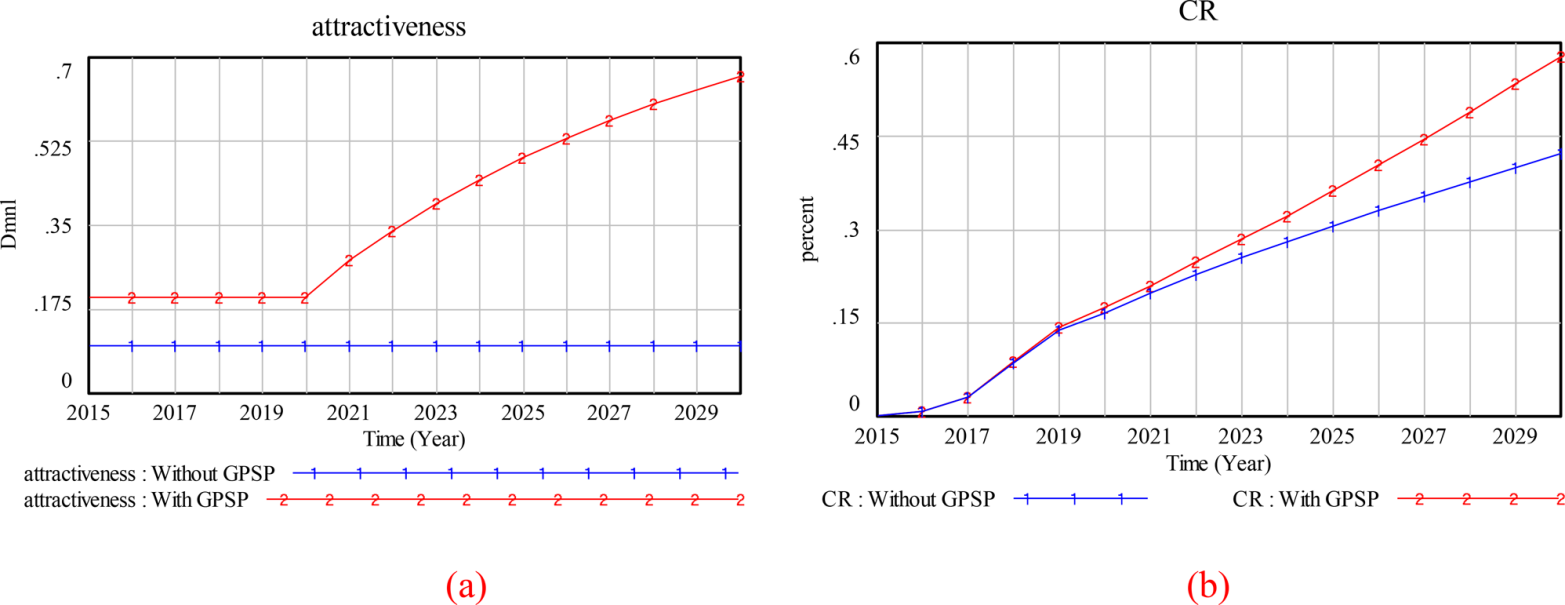
Comparison of (**a**) attractiveness and (**b**) contract rate, with and without the GPSP (GP service package)

To investigate the dynamics of the contract rate for 2015–2030, we established two scenarios, one incorporating the GP service package and one without. The results unequivocally demonstrated a substantial increase in the attractiveness of GP contract service to the occupational population when the GP service package is integrated into the model, particularly for 2020–2030 (Fig. 4(a)). The rapid rise in attractiveness can be attributed to the introduction of GP-building services in 2020. The findings strongly suggest that the GP service package plays a pivotal and beneficial role in enhancing attractiveness. Moreover, the package influences the contract rate among the occupational population. In the scenarios with and without the GP service package, the predicted contract rates reach 57.8% and 42.3%, respectively, by 2030 (Fig. 4(b)). Thus, a 15.4% increase in the contract rate is attributable to the GP service package intervention.

### Combined effects of the GP service package and complementary incentive policies

**Fig. 5:**
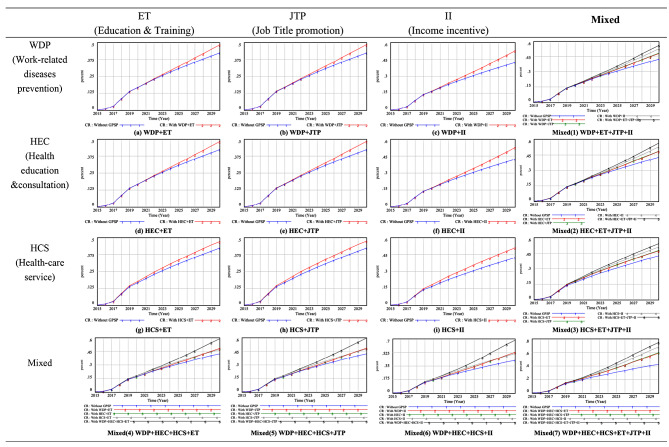
Mixed effects of supply-side factors (ET, JTP, II) and demand-side factors (WDP, HEC, HCS) on the contract rate

Demand- and supply-side factors influence the effects of the GP service package and the complementary incentive policies on the contract rate, and simulations analyzed the combined impact of these factors. When the package included only WDP and ET or JTP interventions, both contract rates were predicted to reach approximately 48% by 2030 (see Fig. 5 (a) and (b)), representing an increase of approximately 6% compared with the “without scenario.” When the GP service package focused solely on WDP and II, the contract rate was expected to increase by 10% in 2030 (see Fig. 5(c), slightly higher than Fig. 5 (a) and (b)). Similar trends were observed when the GP service package included only HEC or HCS in combination with the three supply-side factors individually, with a maximum contract rate of about 52.5% by 2030. Notably, II have a more significant impact on the contract rate than JTP and ET, resulting in a 3–5% higher contract rate (Fig. 5(a)–(i)).

The demand-side factors, WDP, HEC, and HCS, contributed to varying degrees in increasing the contract rate, with a marginal difference of approximately 2% (Fig. 5(a)–(i)). When all three supply-side factors were offered and only one demand-side factor, the contract rate was predicted to be around 54% by 2030 (Fig. 5: Mixed 1–3). Similarly, when all three demand-side factors were provided and a single supply-level factor, the contract rate was expected to reach 58–68% by 2030 (see Fig. 5: Mixed 4–6). Clearly, the demand-side factors had a more significant effect in increasing the contract rate. The combined effects of the six supply- and demand-side factors yielded a significant increase of approximately 33.1% in the contract rate compared with the scenario without the GP service package (Fig. 5: Mixed 7).

**Fig. 6:**
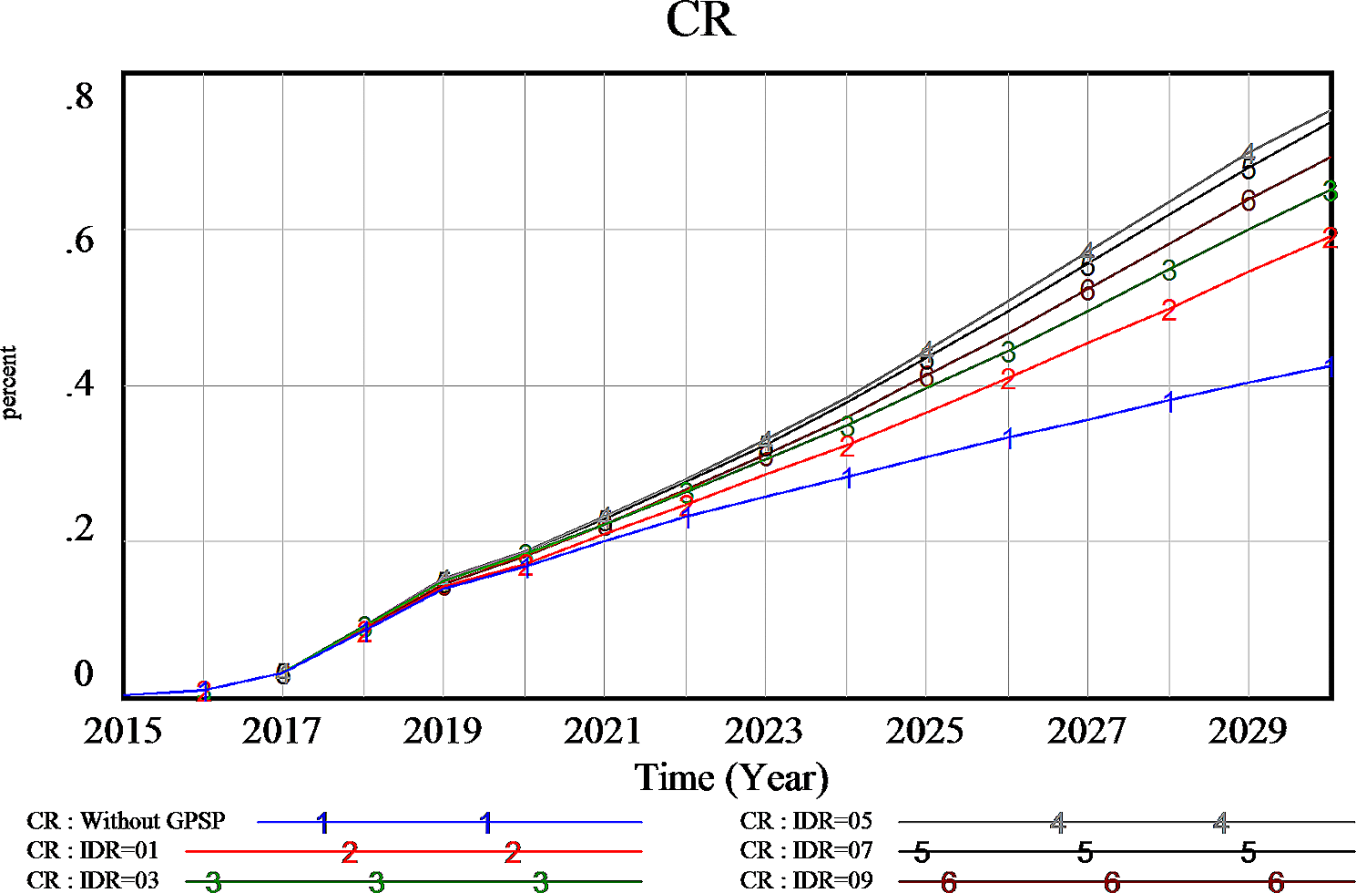
The influence of the income distribution ratio on the contract rate

The income distribution ratio (IDR), a metric for assessing the proportion of income allocated to GPs, influences the driving force of the GP team and provides a measure of income inequality within the team. Five income distribution scenarios were considered, ratios of 0.1, 0.3, 0.5, 0.7, and 0.9, to examine the impact on the contract rate. The simulation results are depicted in Fig. 6. We observed that a higher income distribution ratio to GPs, the core members of the team, resulted in higher contract rates than packages with higher income distribution to other GP team members. For instance, an income distribution ratio of 0.7 led to a 9% higher contract rate than a 0.3 income distribution ratio did. Nevertheless, the highest contract rate of 75.2% for the occupational population in 2030 was observed at an income distribution ratio of 0.5, followed closely by a ratio of 0.7, and there was a 2% difference in the 2030 contract rates between these two distribution ratios. These results underscore the significance of a relatively equal income distribution within the GP team, as it positively correlates with a higher contract rate.

## Discussion

The simulation results of the system dynamic model indicated that the GP service package had a favorable impact, increasing the contract rate of the occupational population. Moreover, the contract rate increased more significantly after the integration of complementary incentive policies in the GP service package. Often, researchers have performed factor analyses, relying on static models that overlook the dynamics and complexity within policy systems [79, 80], revealing that factors such as personal health state, medical skills, individual cognition are key influences affecting contract behavior [80–82]. In addition, the contract rate has been analyzed in terms of the factors influencing GP motivation, which can be categorized into internal motivation (professional belonging, occupational motivation, occupational efficacy) and external motivation (skills development, rewards, promotion) [83]. In contrast, this paper proposed a system dynamics model to test the effect of GP service package and complementary incentive policies on both the demand and the supply side, exploring the combined effect to identify the most effective solution to address limited health management for the occupational population. This model offers a systematic and comprehensive approach to policy formulation, constructs a realistic policy network, and provides an intuitive means to assess the outcomes of various policies on the contract rate, yielding a net effect assessment.

The occupational population experiences suboptimal health states as a result of stress, sedentary work, and insufficient exercise, but this often remains unnoticed because obvious disease symptoms are absent [84]. In response, the Healthy China Action Plan 2019–2030 advocates protective actions to reduce or prevent work-related skeletal system disorders and stress [85]. Shanghai encourages individuals in functional communities to contract with GP teams to improve their health outcomes. However, these initiatives are in the pilot stage and confront practical challenges [25]. Our study identified three factors of GP service packages, i.e., WDP, HEC, and HCS, that contributed significantly to the overall appeal of such packages to the occupational population. This study suggests that the impact of these demand factors surpasses that of the supply-side factors, which we have not thoroughly explored in existing research. The findings of our demand-side analysis emphasize the importance of policy interventions that prioritize the development of demand-oriented and targeted service packages designed for the occupational population. Such measures have the potential to significantly improve the overall health and well-being of the occupational population.

From a supply-side perspective, II, JTP, and ET had a positive impact on the contract rate, with II being the most sensitive of these three factors. Research has revealed the positive effect of II in increasing the quantity of primary care services, elevating patient visit rates, improving care continuity, and promoting adherence to recommended visit frequencies [83, 86]. Several countries have implemented incentive programs to motivate medical staff to provide continuous and higher quality services, including the pay-for-performance scheme of the United Kingdom [87], the Health Care Homes trial in Australia [88], and the Patient-Centered Medical Home initiative in the United States [89]. China has proposed guidelines to establish a reasonable incentive policy and enhance the quality of contract services [90]. We found that JTP and ET have a positive effect on work motivation among GPs, leading to improvements in service quality and a higher contract rate. Studies have emphasized that this influence pathway primarily enhances the sense of value among GP team members, thereby increasing job satisfaction and work motivation [91, 92]. 

We found that income distribution, rather than income, played a pivotal role in determining the contract rate, as the provision of a contract service is a collaborative endeavor carried out by the GP and their team members. In Shanghai, each GP team consists of at least one GP, one community nurse, one public health physician, and a rehabilitation therapist. The team collaboratively provides comprehensive services [93], with GPs mainly handling primary diagnosis, referrals, and visiting services. Community nurses assist in daily diagnosis, treatment, and health management. Public health physicians oversee public health service management, guidance, and quality control for the contracted population [94]. The literature has acknowledged the effectiveness of payment mechanisms as a tool for health systems to achieve optimal outcomes, but a specific analysis of the appropriate proportions for the team income distribution has not been conducted [95]. In this study, we identified an allocation ratio of 0.5 as an optimal incentive share for the GP team, which is a fairer income distribution proposal than the current practice. The logic behind this allocation ratio is a more extensive and in-depth collaboration among primary health-care workers to achieve a better integrated primary health system. This finding suggests a basis for future practical decision-making on the income distribution.

## Conclusion

The system dynamics model developed in this paper for GP contract services targeting occupational groups offers several advantages. First, it fills a crucial gap in the current health management practices, which primarily focus on the elderly and children, by specifically addressing the needs of the occupational population. Second, we proposed a GP service package and complementary incentive policies, incorporating them into the system for quantitative examination. We found that an occupational-population-centered GP service package, combined with complementary incentive policies, matters in terms of achieving significant increases in the contract rate because it increases the attractiveness of GP services and motivates primary health-care workers. We identified the income distribution as the most sensitive of the factors considered, and found that 50% was the optimal ratio. This systematic research approach captures the dynamic and complex nature of policy systems, providing a valuable perspective for policy formulation and intervention in GP contract services for occupational groups. However, it is important to acknowledge that there may be underlying factors that have not been fully explored or revealed in this study. By addressing the specific needs and challenges of this population, future research can inform evidence-based strategies that optimize health outcomes and improve the overall well-being of the occupational population.

### Electronic supplementary material

Below is the link to the electronic supplementary material.

Supplementary Material 1

## Data Availability

The data that support the findings of this study are available from the corresponding author upon reasonable request.

## Abbreviations

GP: general practitioner
GPSP: general practitioner service package
WDP: work-related disease prevention
HEC: health education & counseling
HCS: health-care service
II: income incentives
JTP: job title promotion
ET: education & training
IDR: income distribution ratio
CR: contract rate
