# Supplementary material for: Does a GP service package matter in addressing the absence of health management by the occupational population? A modelling study
Source: BMC Health Serv Res. 2024 May 17;24:638. doi: 10.1186/s12913-024-10954-9 (PMC11100196; doi:10.1186/s12913-024-10954-9)
Supplement: Supplementary file 1 — Supplementary Material 1 [file 12913_2024_10954_MOESM1_ESM.docx]

Appendix 1

A study on the perception and needs assessment of contract family doctors among young and middle-aged population

# Part 1: Basic Information

**A1** Birth year _______

**A2** Gender ① Male ② Female

**A3** Ethnicity: ①Han ② Zhuang ③ Hui ④ Uyghur ⑤ Other

**A4** Domicile: ① Shanghai ② Non-Shanghai

**A5** Place of residence: ① North Bund Street, Hongkou District ② Other Streets, Hongkou District③ Huangpu ④ Xuhui ⑤ Pudong New District ⑥ Changning ⑦ Jing'an ⑧ Putuo ⑨ Chongming ⑩ Yangpu ⑪ Minhang ⑫ Baoshan ⑬ Jiading ⑭ Jinshan ⑮ Songjiang ⑯ Qingpu ⑰Fengxian

**A6** Employment status: ① Employer ② State-owned enterprise or collective state enterprise employee ③ Private enterprise employee ④ Joint venture employee ⑤ other

**A7** Job: ① The state unit, party and group organization, business person in charge of the state unit ② Professional technicians ③ Clerical personnel and relevant④ Business, service personnel ⑤ Others

**A8** Total annual revenue (gross wage or operating income): _____ million yuan

**A9** Highest education: ① Junior high school and below ② High school ③ Junior college ④Undergraduate ⑤ Postgraduate

**A10** Marital status is:① unmarried ② Unmarried cohabiting ③ First marriage ④ Remarriage ⑤ Divorced ⑥ Widowed ⑦ Other

**A11** Do you have social health insurance (excluding commercial): ① Yes ② None (if yes, continue, if no, skip to A13)

**A12** If yes, which of the following social medical insurance? ①Basic medical insurance for urban workers ② Basic medical insurance for urban and rural residents

**A13** You have ____ people in your family with____ generations.

**A14** Do you think your class belongs to: ① Very low ② Low ③ Middle ④ High ⑤ Extremely high

**A15** Do you think the class of your parents belongs to: ① Very low ② Low ③ Middle ④ High ⑤ Extremely high

# Part 2: Family Doctor Building Services

**B1** Have you ever heard of Family Doctor Building service? ① Not familiar ② Not very familiar ③ General ④ Familiar ⑤ Very familiar

**B2** Do you understand the contents of the family doctor building service? ① Not familiar ② Not very familiar ③ General ④ Familiar ⑤ Very familiar

**B3** Have you made use of family doctor building services? ① Yes ② No (If no, jump to B6)

**B4** Which family doctor building services have you used? (Multiple choices for this question)

① Health promotion (Winter disease summer treatment, medicine tea, traditional Chinese medicine to lose weight ② Health examination (oral health examination) ③ Health consultation ④ Physiotherapy services (acupuncture, massage physiotherapy services, etc.) ⑤ Traditional Chinese medicine and western medicine consultation ⑥ Pharmacy service ⑦ Other

**B5** Please rate building service satisfaction? ① 1 ② 2 ③ 3 ④ 4 ⑤ 5

**B6** Please serve the family doctor according to your own needs on a score of 1-5, 1 means not needed, 5 means very needed. The higher the score, the more you need it.

| Demand content | Specific item | Score |
| --- | --- | --- |
| Collaborative services of specialized hospitals | B6-1 Upward referral: After diagnosis by a family doctor, it is promptly transferred to a specialist in a large hospital |  |
|  | B6-2 Downward referral: Hospital discharge transferred back to the community for follow-up or health management by the family doctor |  |
|  | B6-3 Specialist consultation: Regularly scheduled consultation by specialists from major hospitals |  |
| Health Management Services | B6-4 Creation, Updating and Informational Inquiry of Health Records |  |
|  | B6-5 Free health examination and consultation: e.g., on-site blood sugar, blood pressure, and lipid checkups |  |
|  | B6-6 Health information promotion |  |
|  | B6-7 Group health seminar and guidance in thematic format |  |
|  | B6-8 Health problem consultation |  |
|  | B6-9 Psychological problems guidance |  |
| Pharmacy  Services | B6-10 Long prescription: 1-2 months supply for chronic and stabilized patients |  |
|  | B6-11 Extended prescription: a prescription previously written in a major hospital can be transferred in a community hospital |  |

**B7** Please rate family doctor building services according to your own needs on a score of 1-5, 1 means not needed, 5 means very needed. The higher the score, the more you need it.

| Demand content |  |
| --- | --- |
| B7-1 Eye disease prevention |  |
| B7-2 Cervical spondylosis/frozen shoulder/mouse hand prevention |  |
| B7-3 Breast/cervical diseases Prevention |  |
| B7-4 Seasonal cold and headache prevention |  |
| B7-5 Chronic gastritis prevention |  |
| B7-6 Sports injury prevention: Avoid physical injury caused by improper exercise |  |
| B7-7 Traditional Chinese Medicine constitution identification |  |
| B7-8 Tui Na/moxibustion/Cupping/Gua Sha/acupuncture |  |
| B7-9 Guidance on physical examination checklist consultation |  |
| B7-10 Body mass Index assessment and fitness guidance |  |
| B7-11 Drug delivery services: After the family doctor prescribing, drugs are uniformly distributed to the building by a third party |  |

**B8** According to your needs, select a way you would most like health building services to be carried out? (Pay attention! Choose a single option)

① Online consulting family doctor family doctor ② Make an appointment with family doctor ③ On-site consulting family doctor④ Health lecture⑤ Physiotherapy experience

**B9** Please select an item of your preferred service hours ① 1 hour before work ② 1 hour at noon ③ 1 hour after work ④ Weekend Time

**B10** If you want to enjoy more services, would you like to sign a free contract with a family doctor and enjoy the contracted services of a family doctor?

① Not willing ② Not very willing ③ General ④ Willing ⑤ Very willing

**B11** If you want to enjoy more personalized service, would you like to purchase a personalized service package for white-collar workers? ① Yes ② No (if no, skip to B1)

**B12** The overall price of the personalized service package for white-collar workers that you are willing to accept is:

① 0 ~ 50 yuan/year ② 50 ~ 100 yuan/year ③100 ~ 150 yuan/year ④150 ~ 200 yuan/year ⑤ 200 yuan/year or more

**B13** Your preferred payment method for this service pack is:

① Medicare individual accounts pay separately ② Individual cash payments separately ③ Medicare and individual co-payment ④ Enterprise contract payment

# Part 3. Utilization of family doctor services

**1. Utilization of community hospitals**

**C1** Where do you usually go first when you get sick? ① Community hospital ② Secondary hospital ③ Tertiary hospital ④ Not necessarily

**C2** The choice of your visit will depend most on: (Pay attention! This question is a single choice)

① Personal habits ② Severity of the disease ③ Whether there are acquaintances ④ Others

**C3** Have you ever been treated in the community health service center (station)? ① Yes ② No (If yes, continue, if no, go to question C6)

**C4** How many times have you visited your community health service center in a year? ① 1-6 times ② 7-12 times ③ 13-24 times ④ more than 25 times

**C5** Do you have contracted with a family doctor: ① Yes ② No

**C6** Please check the community services you have utilized (Multiple choices for this question) ① Basic medical services ② Referral service ③ Dispensing service ④ Long-term prescription services ⑤ Extended prescription service ⑥ Health records-related services ⑦ Health education service ⑧ Health examination service ⑨ Health advisory services ⑩ Chronic disease tracking service ⑪ Rehabilitation guidance ⑫ Home-based service ⑬ Family bed service

**2. Community hospital referral situation**

**C7** Do you know about "two-way referral"? ① Yes ② No (not clear to select no)

**C8** Do you usually go directly to secondary and tertiary hospitals or are you referred through family doctor? ① Go directly to secondary and tertiary hospitals ② Family doctor referral (if yes, continue, no jump to question D1)

**C9** Under what circumstances were you referred:① Recommended by the attending physician ② At your own request ③ Both

**C10** Were you referred to the hospital you wanted? ① Yes ② No

**C11** Was the referral process smooth? ① Yes ② No

**C12** After being successfully transferred to a superior medical institution, is it transferred back to a subordinate medical institution after receiving medical services? ① Yes ② No

**C13** Your satisfaction with the service during the referral process: ① Very dissatisfied ② Not very satisfied ③ General ④ Satisfied ⑤ Very satisfied

**C14** Do you receive specialist treatment from a superior hospital? ① Yes ② No

# Part 4. Satisfaction Degree

**D1** Have you used any community health services? ① Yes ② No (if yes, continue, if no, end the questionnaire)

**D2** If yes, please rate the overall satisfaction with community health services ________ (0-10 points)

**D3** If yes, please rate your community health services on the following (0-10)

| External perception | Score | Value judgment | Score | Service content | Score |
| --- | --- | --- | --- | --- | --- |
| A: Medical environment |  | H: Reasonable prescription |  | O: Long prescription |  |
| B: Facilities and equipment |  | I： Reasonable inspection |  | P: Extension prescription |  |
| C: Drug equipment |  | J: Reasonable charges |  | Q: Traditional Chinese Health Care |  |
| D: Number of medical staff |  | K: Accurate diagnosis |  | R: Health check-up |  |
| E: Service capability |  | L: Effective treatment |  | S: Health Education |  |
| F: Service attitude |  | M: Effective communication |  | T: Family bed |  |
| G: Service efficiency |  | N: Effective health management |  | U: Referral service |  |

**D4** Do you think family doctors are able to meet basic needs? ① There is a big gap ② Not satisfied yet ③ General ④ Satisfied ⑤ Fully satisfied

**D5** Do you think family doctors meet individual needs? ① There is a big gap ② Not satisfied yet ③ General ④ Satisfied ⑤ Fully satisfied

**D6** What you would like to see further improved in the family doctor building service: ① More convenient to dispense medicine ② More flexible service time ③ More diversified service methods④ Other

Appendix 2

Survey on Work Stress and Appeal for Rights and Interests of Medical Staff

(Including primary medical staff and medical staff in secondary and tertiary hospitals)

# A: Basic demographic information

**A1** Birth year _______

**A2** Gender ① Male ② Female

**A3** Marital status ① Unmarried ② Married ③ Divorced ④ Widowed ⑤ Other

**A4** Education level ① High school (Technical secondary school) or below ② Bachelor's degree (Junior college) ③ Master's degree ④ Doctor's degree

**A5** Place of residence ① This city's street ② Other streets in this city ③ Other provinces and cities

**A6** Major ① General medicine ② Clinical medicine ③ Public health ④ Nursing ⑤ Other

**A7** What is your occupation category? ① Doctors ② Nurses ③ Public health physicians ④ Technicians ⑤ Others

**A8** Title ① No ② Junior ③ Intermediate ④ Deputy senior ⑤ Senior

**A9** You have been practicing medicine for ___years and have worked in this organization for ___ years

**A10** Your department belongs to: ① General practice ② Internal medicine ③ Surgery ④ Obstetrics and Gynecology ⑤ Pediatrics ⑥ Stomatology ⑦ Medical technology ⑧ Other

**A11** Your hospital belongs to: ① Community health service center (site) ② Secondary hospital ③ Tertiary hospital

**A12** Your location: ① Huangpu, ② Xuhui, ③ Changning, ④ Jing 'an, ⑤ Putuo, ⑥ Hongkou, ⑦ Yangpu, ⑧ Pudong, ⑨ Minhang, ⑩ Baoshan, ⑪ Jiading, ⑫ Jinshan, ⑬ Songjiang, ⑭ Qingpu, ⑮ Fengxian ⑯ Chongming

# B: Work pressure

**B1** How is your overtime situation at work: ① Never work overtime ② Rarely work overtime ③ Occasionally work overtime ④ Often work overtime ⑤ Almost every day

**B2** How is your usual work intensity: ① Very weak ② Relatively not strong ③ General ④ Relatively strong ⑤ Very strong

**B3** Stress level of current workload: ① No pressure at all ② Relatively no pressure ③ General ④ Relatively stressful ⑤ Very stressful

**B4** The impact of family doctor contracting services in the community on your workload:

① Greatly reduce the workload ② Slightly reduce the workload ③ No impact ④ Slightly increase the workload ⑤ Significant increase the workload

**B5** The impact of COVID-19 prevention on your workload:

① Greatly reduce the workload ② Slightly reduce the workload ③ No impact ④ Slightly increase the workload ⑤ Significant increase the workload

**B6** Do you have adequate personal safety protection during COVID-19 prevention work?

① Serious lack of personal protection ② Lack of personal protection ③ General ④ Personal protection is better ⑤ Personal protection is very good

**B7** Personal anxiety at the beginning of the COVID-19 outbreak:

① Very anxious ② Relatively anxious ③ General ④ Relatively not anxious ⑤ Not anxious at all

**B8** Personal anxiety after the normalization of the COVID-19 outbreak:

① Very anxious ② Relatively anxious ③ General ④ Relatively not anxious ⑤ Not anxious at all

**B9** The support coordination from the team:

① Very weak ② Relatively weak ③ General ④ Relatively strong ⑤ Very strong

**B10** The support from other departments in your organization:

① Very weak ② Relatively weak ③ General ④ Relatively strong ⑤ Very strong

**B11** The support from government:

① Very weak ② Relatively weak ③ General ④ Relatively strong ⑤ Very strong

**B12** Self-assessment of health status

① Very poor ② Relatively poor ③ General ④ Fairly good ⑤ Very good

# C Appeal for Rights and Interests and satisfaction

**C1** Which of the following rights do you most need?

| Rights and interests appeal | No need | Not really need | General | Need | Very need |
| --- | --- | --- | --- | --- | --- |
| Raise pay |  |  |  |  |  |
| Routine reimbursement |  |  |  |  |  |
| Housing subsidy |  |  |  |  |  |
| Staff recruitment |  |  |  |  |  |
| Children education |  |  |  |  |  |
| Supplementary provident fund |  |  |  |  |  |
| Training and further study |  |  |  |  |  |
| Academic promotion |  |  |  |  |  |
| Appointment of professional title |  |  |  |  |  |
| Paid leave |  |  |  |  |  |
| Recreational activities |  |  |  |  |  |
| Feedback channel |  |  |  |  |  |

**C2** Your current average annual after-tax income (including bonuses, allowances, etc.) is about _________ million yuan.

**C3** Do you think your efforts and income match:

① Not match ② Relatively not match ③ General ④ Relatively match ⑤ Very match

**C4** Do you think the current salary can motivate you to carry out work:

① Not at all ② Relatively not, ③ General ④ Relatively capable, ⑤ Completely capable

**C5** Do you think your current job has career prospects:

① Not at all ② Relatively none ③ General ④ Relatively yes ⑤ Very yes

**C6** Your degree of burnout:

① I have no burnout at all ② I rarely feel burnout ③ I have at least one burnout

④ My job burnout is serious ⑤ My job burnout is very serious

**C7** Are you considering or have you ever considered a job change: ① Yes ② No

**C8** Please rate your satisfaction with the following aspects on a score of 1-5 (the higher the better, 5 being very satisfied)

| Item | Very dissatisfied | Less satisfied | General | Relatively satisfied | Very satisfied |
| --- | --- | --- | --- | --- | --- |
| Work environment |  |  |  |  |  |
| Medical equipment |  |  |  |  |  |
| Information system |  |  |  |  |  |
| Revenue and performance |  |  |  |  |  |
| Organization management |  |  |  |  |  |
| Team building |  |  |  |  |  |
| Division of labor and cooperation |  |  |  |  |  |
| Promotion of professional title |  |  |  |  |  |
| Continuing training |  |  |  |  |  |
| Spiritual and cultural activities |  |  |  |  |  |
| Overtime mechanism |  |  |  |  |  |
| Leave system |  |  |  |  |  |
| Central support collaboration |  |  |  |  |  |
| Feedback and complaint mechanism |  |  |  |  |  |
| Department or team support collaboration |  |  |  |  |  |
| Policy support |  |  |  |  |  |

Appendix 3

1. The development of General Practitioner Service Package (GPSP)
2. The Preliminary Development of the Service Package Based on Pre-survey. The research team held discussions with the directors of community health service centers, medical departments, family doctor teams, and relevant personnel from the Community Health Service Management Center. They organized discussions with white-collar workers in office buildings and developed the "Questionnaire on the Service Demand of Family Doctors in Buildings for Middle-aged and Young People." Based on the preliminary survey results, the "Family Doctor Building Service Contracted Service Package" was formulated and adjusted according to the demand-side investigation covering three regions in Shanghai. The actual survey covered a total of 2366 samples in Shanghai, with 2272 valid responses, achieving an effective rate of 96.03%. The specific questionnaire content is provided in Appendix 1.
3. Exploratory factor analysis. Exploratory factor analysis was employed to capture the attractiveness factors of contracted services, incorporating 24 indicators from D601 to D712. The principal component method was utilized for factor extraction, followed by orthogonal axis rotation through Varimax to maximize variance. The scree plot of the exploratory factor analysis is depicted in Supplementary Figure 1.


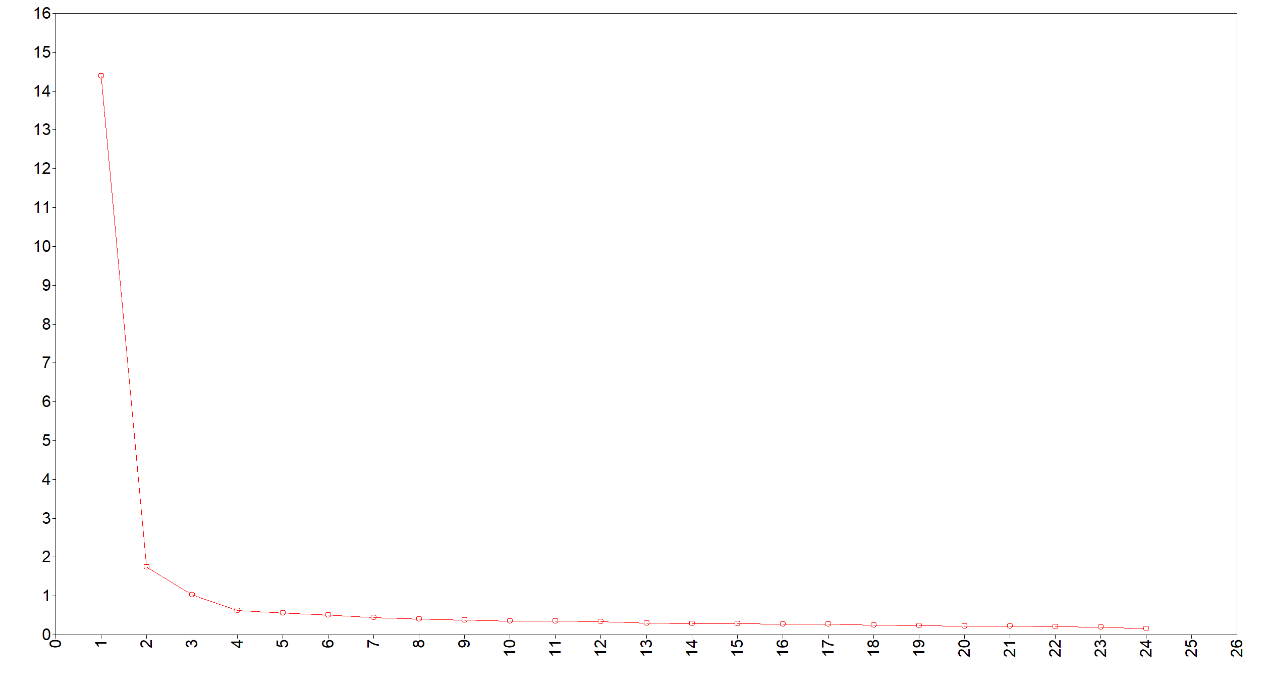


Supplementary Figure 1 Exploratory analysis of demand appeal factors - gravel diagram

1. The fitting parameters and rotated factor loadings of the three models are presented in Supplementary Tables 1 and 2, respectively. The structure of the four-factor model exhibits considerable complexity, with five factors displaying cross-loadings. From the perspective of model fit indices, the choice between the three-factor and four-factor models is challenging. However, the scree plot supports retaining a three-factor structure. Consequently, considering both fit indices and graphical representation, the decision is made to retain the three-factor model.

Supplementary Table 1 Demand exploratory factor analysis model fit index

| Model | c^2^ | *df* | TLI | CFI | AIC | BIC | SRMR | RMSEA (90% CI) |
| --- | --- | --- | --- | --- | --- | --- | --- | --- |
| two-factor | 2099.64*** | 229 | 0.896 | 0.914 | 139426.84 | 139965.71 | 0.034 | 0.062 (0.059, 0.064) |
| three-factor | 1237.76*** | 207 | 0.937 | 0.953 | 137711.04 | 138374.70 | 0.021 | 0.048 (0.046, 0.051) |
| four-factor | 801.21*** | 186 | 0.958 | 0.972 | 136847.33 | 137191.66 | 0.017 | 0.039 (0.036, 0.042) |

Note: *** p < 0.001

Supplementary Table 2 Demand Exploratory Factor Loading Matrix

| Content | two-factor | | three-factor | | | four-factor | | | |
| --- | --- | --- | --- | --- | --- | --- | --- | --- | --- |
|  | F1 | F2 | F1 | F2 | F3 | F1 | F2 | F3 | F4 |
| D601 | **0.81*** | -0.04 | **0.86*** | 0.01 | -0.24* | **0.84*** | 0.03 | -0.03 | 0.03 |
| D602 | **0.86*** | -0.05 | **0.92*** | -0.03 | -0.22* | **0.87*** | -0.01 | 0.01 | 0.04* |
| D603 | **0.77*** | -0.01 | **0.81*** | 0.00 | -0.15* | **0.69*** | 0.03 | 0.11* | -0.03 |
| D604 | **0.76*** | 0.08 | **0.85*** | -0.06 | 0.03 | **0.58*** | -0.05* | **0.33*** | 0.05* |
| D605 | **0.71*** | 0.10* | **0.78*** | 0.01 | -0.02 | **0.54*** | 0.04 | 0.28* | -0.03 |
| D606 | **0.69*** | 0.12* | **0.74*** | 0.04 | -0.01 | **0.49*** | 0.07* | **0.31*** | -0.09* |
| D607 | **0.58*** | 0.28* | **0.71*** | 0.03 | 0.20* | **0.32*** | 0.03 | **0.51*** | 0.04 |
| D608 | **0.49*** | **0.38*** | **0.64*** | 0.06 | 0.30* | 0.17* | 0.06 | **0.64*** | 0.04 |
| D609 | **0.42*** | **0.41*** | **0.63*** | -0.01 | **0.43*** | 0.02 | 0.01 | **0.82*** | -0.01 |
| D610 | **0.45*** | **0.39*** | **0.63*** | 0.02 | **0.35*** | 0.12* | 0.03 | **0.69*** | 0.03 |
| D611 | **0.40*** | **0.43*** | **0.61*** | -0.02 | **0.47*** | -0.04 | 0.001 | **0.91*** | -0.05* |
| D612 | **0.39*** | **0.46*** | **0.61*** | 0.01 | **0.46*** | 0.02 | 0.01 | **0.81*** | 0.06* |
| D701 | -0.01 | **0.82*** | -0.03 | **0.83*** | 0.02 | -0.04 | **0.79*** | 0.06 | 0.03 |
| D702 | 0.04 | **0.76*** | -0.03 | **0.89*** | -0.12* | 0.04 | **0.88*** | -0.07 | -0.10* |
| D703 | 0.06 | **0.76*** | 0.02 | **0.82*** | -0.06* | 0.05 | **0.79*** | -0.003 | -0.02 |
| D704 | -0.02 | **0.86*** | -0.05 | **0.89*** | 0.002 | -0.05* | **0.86*** | 0.05 | -0.001 |
| D705 | 0.02 | **0.80*** | -0.01 | **0.83*** | -0.01 | -0.02 | **0.80*** | 0.07 | -0.02 |
| D706 | 0.002 | **0.82*** | -0.01 | **0.82*** | 0.03 | -0.01 | **0.75*** | 0.04 | 0.12* |
| D707 | 0.07* | **0.74*** | 0.02 | **0.81*** | -0.06 | 0.04 | **0.77*** | 0.01 | -0.03 |
| D708 | 0.14* | **0.65*** | 0.07 | **0.79*** | -0.15* | 0.15* | **0.77*** | -0.09 | -0.04 |
| D709 | 0.08* | **0.78*** | 0.09* | **0.74*** | 0.06 | 0.07* | **0.67*** | 0.06 | 0.20* |
| D710 | -0.04 | **0.85*** | -0.02 | **0.78*** | 0.11* | -0.03 | **0.69*** | 0.05 | 0.29* |
| D711 | -0.01 | **0.87*** | 0.02 | **0.77*** | 0.13* | 0.04* | **0.68*** | -0.04* | **0.50*** |
| D712 | -0.03 | **0.86*** | 0.01 | **0.75*** | 0.15* | -0.02 | **0.65*** | 0.06 | **0.40*** |
|  | Factor Correlation Matrix | | | | | | | | |
| F2 | 0.71* |  | 0.79* |  |  | 0.65* |  |  |  |
| F3 |  |  | 0.25 | 0.47* |  | 0.67* | 0.79* |  |  |
| F4 |  |  |  |  |  | 0.18* | 0.27* | 0.38* |  |

Note: Loadings greater than 0.3 are bolded; * p < 0.05

1. Model Modification. The initial model fit to the data was not ideal (see Supplementary Table 3), so it is necessary to modify the model. Referring to the Modification Index (MI) reported by the software, and considering theoretical and logical feasibility, the model was modified. The modification process includes allowing errors between items D712 and D711 to be correlated, allowing factor 3 and item D608 errors to be correlated, allowing errors between items D708 and D707 to be correlated, and allowing errors between items D602 and D601 to be correlated. Confirmatory factor analysis results show that the initial three-factor CFA is 3730.01 (P < 0.001), and the modified three-factor CFA is 2552.92 (P < 0.001).

Supplementary Table 3 Confirmatory factor analysis model fit index of attractiveness of workplace services

| Model | c^2^ | *df* | TLI | CFI | AIC | BIC | SRMR | RMSEA (90% CI) |
| --- | --- | --- | --- | --- | --- | --- | --- | --- |
| The initial three-factor CFA | 3730.01*** | 249 | 0.914 | 0.922 | 138810.58 | 139236.00 | 0.038 | 0.081 (0.078, 0.083) |
| The revised three-factor CFA | 2552.92*** | 245 | 0.942 | 0.949 | 137641.48 | 138089.59 | 0.034 | 0.066 (0.064, 0.069) |

Note: *** p < 0.001

1. The estimated results of the modified three-factor model are presented in Supplementary Table 4. Factor 1 (f1) includes items D601 to D608, named as Health care Services; Factor 2 (f2) includes items D701 to D712, named as Work-related Diseases Prevention; Factor 3 (f3) includes items D609 to D612, named as Health Education & Consultation.

Supplementary Table 4 Estimation results of the revised three-factor CFA model (standardized)

| Factors | Items | Estimate（S.E.） |
| --- | --- | --- |
| f1- Health care Services | D601: long prescription | 0.748***(0.011) |
|  | D602: Extended prescription | 0.787***(0.009) |
|  | D603: Drug dispensing service/drug delivery services | 0.755***(0.010) |
|  | D604: Upward referral | 0.816***(0.008) |
|  | D605: Downward referral | 0.778***(0.010) |
|  | D606: Specialist consultation | 0.781***(0.009) |
|  | D607: Establishing health records | 0.807***(0.009) |
|  | D608: Health examination | 0.789***(0.009) |
| f2- Work-related Diseases Prevention | D701: Eye disease prevention | 0.817***(0.008) |
|  | D702: Cervical spondylosis/frozen shoulder/mouse hand prevention | 0.800***(0.008) |
|  | D703: Breast/cervical diseases Prevention | 0.810***(0.008) |
|  | D704: Seasonal cold and headache prevention | 0.857***(0.006) |
|  | D705: Chronic gastritis prevention | 0.828***(0.007) |
|  | D706: Sports injury prevention | 0.822***(0.008) |
|  | D707: Traditional Chinese Medicine | 0.785***(0.009) |
|  | D708: Tui Na/moxibustion/Cupping/Gua Sha/acupuncture | 0.756***(0.010) |
|  | D709: Guidance on physical examination checklist consultation | 0.837***(0.007) |
|  | D710: Body mass Index assessment and fitness guidance | 0.818***(0.008) |
|  | D711: Open use of smart health devices | 0.842***(0.007) |
|  | D712: Accessibility and visibility of personal digital health records | 0.815***(0.008) |
| f3- Health Education & Consultation | D609: Health information promotion | 0.845***(0.007) |
|  | D610: Group health seminar | 0.817***(0.008) |
|  | D611: Health problem consultation | 0.846***(0.007) |
|  | D612: Psychological problems guidance | 0.862***(0.007) |

1. After completing the model validation and correction, a higher-order factor model fit was conducted. The results of the higher-order factor model fit are presented in Supplementary Table 5. According to the simulation results, c^2^ is 2552.92 (P<0.001), and fit indices, including TLI (0.942), AIC (137641.48), BIC (138089.59), CFI (0.949), RMSEA (0.066), SRMR (0.034), generally meet the standard values.

Supplementary Table 5 Fitting results of high-order factor model

| Model | c^2^ | *df* | TLI | CFI | AIC | BIC | SRMR | RMSEA (90% CI) |
| --- | --- | --- | --- | --- | --- | --- | --- | --- |
| Second-order model | 2552.92*** | 245 | 0.942 | 0.949 | 137641.48 | 138089.59 | 0.034 | 0.066 (0.064, 0.069) |

Note: *** p < 0.001

2. The development of complementary incentive policies

1. During the questionnaire design phase, the research team collaborated with the family doctor teams from partner institutions to develop a survey specifically addressing the primary demands of grassroots medical staff. The survey encompassed various aspects of healthcare professionals' occupational demands, including raise pay, routine reimbursement, housing subsidy, staff recruitment, child education, supplementary provident fund, training and further study, academic promotion, appointment of professional title, paid leave, recreational activities, feedback channel (see the questionnaire in the appendix). The research team further conducted factor identification and structural relationship construction for these 12 demand items. The exploratory factor analysis for healthcare professionals' rights and demands is illustrated in the scree plot in Supplementary Figure 2.


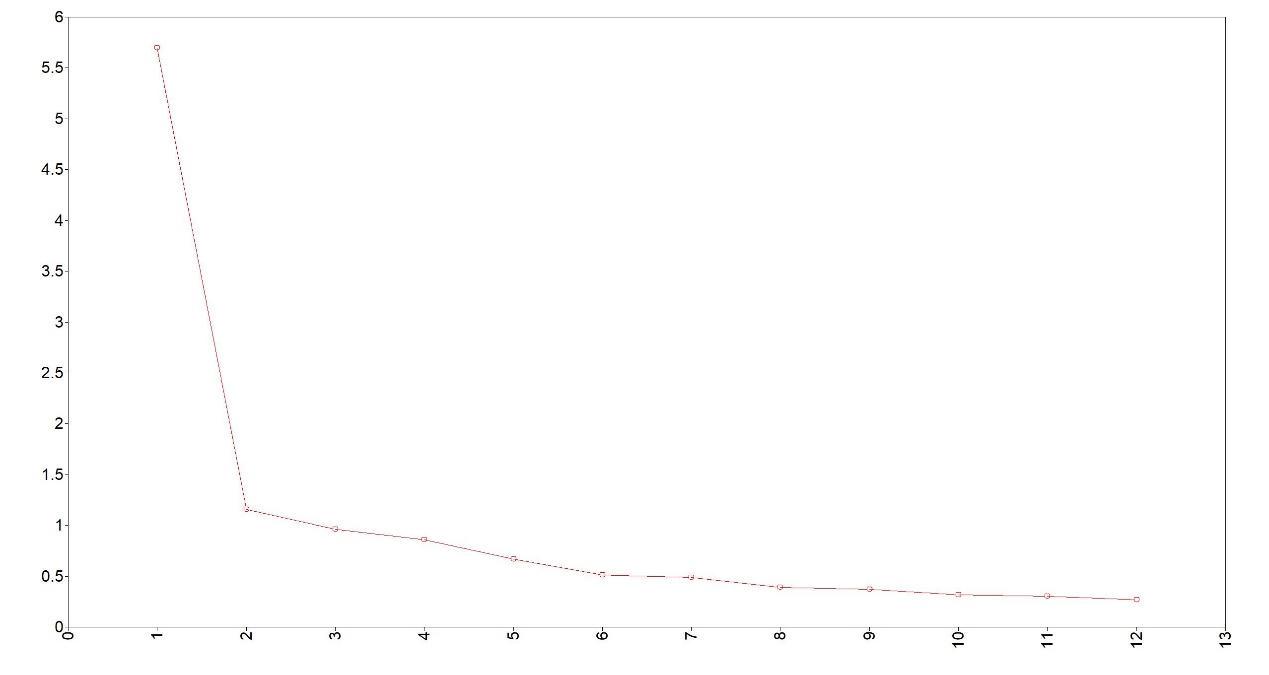


Supplementary Figure 2 Exploratory analysis of medical staff's rights and interests appeal factors-crushed stone chart

1. The fitting parameters and rotated factor loadings of the three models are presented in Supplementary Tables 6 and 7, respectively. The two-factor model's fit indices are near the critical values, suggesting that Item 9 has almost equal loadings on both factors. The fit indices for the three-factor model show significant improvement compared to the two-factor model, and the factor loading matrix indicates that Items 5, 6, 9, 10, 11, and 12 are distinct from one of the factors in the two-factor model. While the four-factor model has the best fit indices, its factor structure is more complex, with four factors exhibiting cross-loadings. From a perspective of structural simplicity, selecting the two-factor and three-factor models is appropriate, and from the viewpoint of model fit indices, choosing the three-factor and four-factor models is suitable. Additionally, the scree plot also supports retaining the three-factor model, so, considering all factors, the three-factor model is selected for retention.

Supplementary Table 6 Fitting index of exploratory factor analysis model for the rights and interests of medical staff

| Model | c^2^ | *df* | TLI | CFI | AIC | BIC | SRMR | RMSEA (90% CI) |
| --- | --- | --- | --- | --- | --- | --- | --- | --- |
| Two-factor | 940.49*** | 43 | 0.789 | 0.863 | 54186.04 | 54449.14 | 0.048 | 0.102 (0.097, 0.108) |
| Three-factor | 570.70*** | 33 | 0.836 | 0.918 | 53537.44 | 53856.53 | 0.034 | 0.090 (0.084, 0.097) |
| Four-factor | 168.45*** | 24 | 0.939 | 0.978 | 53089.93 | 53459.39 | 0.017 | 0.055 (0.047, 0.063) |

Note: *** p < 0.001

Supplementary Table 7 Exploratory Factor Loading Matrix of Medical Staff's Rights and Interests Appeal

| Content | Two-factor | | Three-factor | | | Four-factor | | | |
| --- | --- | --- | --- | --- | --- | --- | --- | --- | --- |
|  | F1 | F2 | F1 | F2 | F3 | F1 | F2 | F3 | F4 |
| 1. Raise pay | **0.79*** | -0.14* | **0.55*** | 0.29* | -0.10 | **0.42*** | -0.02 | **0.43*** | 0.06 |
| 2. Routine reimbursement | **0.65*** | 0.001 | **0.52*** | 0.18* | 0.06 | **0.59*** | 0.01 | 0.02 | 0.23* |
| 3. Housing Subsidies | **0.77*** | -0.10 | **0.90*** | -0.03 | -0.002 | **0.68*** | 0.01 | **0.34*** | -0.06* |
| 4. Staff Recruitment | **0.37*** | 0.16* | **0.36*** | 0.01 | 0.24* | **0.43*** | 0.19* | -0.07* | 0.11* |
| 5. Children Education | **0.43*** | 0.22* | 0.17* | **0.33*** | 0.18* | 0.01 | **0.31*** | **0.47*** | 0.002 |
| 6. Supplementary Provident Fund | **0.63*** | 0.11* | 0.27* | **0.45*** | 0.06 | 0.03 | 0.23* | **0.68*** | 0.02 |
| 7. Training and Further Study | 0.03 | **0.84*** | -0.01 | 0.15* | **0.75*** | -0.03 | **0.80*** | 0.10* | 0.03 |
| 8. Academic Promotion | -0.01* | **0.81*** | 0.04 | -0.003 | **0.82*** | 0.08* | **0.84*** | -0.05* | -0.02 |
| 9. Appointment of Professional Title | **0.40*** | **0.37*** | 0.02 | **0.48*** | 0.27* | -0.02 | **0.39*** | 0.29* | 0.20* |
| 10. Paid Leave | **0.75*** | 0.003 | 0.003 | **0.93*** | -0.19 | 0.003 | -0.03 | **0.40*** | **0.55*** |
| 11. Recreational Activities | **0.56*** | 0.20* | -0.14 | **0.87*** | -0.004 | -0.01 | 0.03 | 0.004 | **0.83*** |
| 12. Feedback Channels | **0.57*** | 0.19* | -0.01 | **0.73*** | 0.04 | 0.12* | 0.07* | 0.01 | **0.68*** |
|  | Factor correlation matrix | | | | | | | | |
| F2 | 0.61* |  | 0.67* |  |  | 0.41* |  |  |  |
| F3 |  |  | 0.42* | 0.62* |  | 0.39* | 0.32* |  |  |
| F4 |  |  |  |  |  | 0.47* | 0.58* | 0.52* |  |

Note: Loadings greater than 0.3 are bolded; * p < 0.05

1. The initial model fit to the data was not ideal (see Supplementary Table 8, so it was necessary to make adjustments to the model. Referring to the modification indices (MI) reported by the software, and considering theoretical foundations and logical feasibility, the model was revised. The modification process included allowing errors between items 12 and 11, allowing errors between factor 3 and item 10, allowing errors between items 5 and 6, and allowing errors between items 9 and 5 to be correlated. After model correction, the chi-square value for the three-factor CFA was 614.18 (P < 0.001), and the fit indices, including TLI (0.929), CFI (0.949), AIC (53427.15), BIC (53667.86), RMSEA (0.066), and SRMR (0.034), generally met the standard criteria, as detailed in Supplementary Table 8.

Supplementary Table 8 Fitting index of confirmatory factor analysis model for medical staff's rights and interest appeal

| Model | c^2^ | *df* | TLI | CFI | AIC | BIC | SRMR | RMSEA (90% CI) |
| --- | --- | --- | --- | --- | --- | --- | --- | --- |
| The initial three-factor CFA | 1129.32*** | 51 | 0.876 | 0.904 | 53934.29 | 54152.61 | 0.048 | 0.103 (0.098, 0.108) |
| The revised three-factor CFA | 614.18*** | 47 | 0.929 | 0.949 | 53427.15 | 53667.86 | 0.040 | 0.078 (0.072, 0.083) |

Note: *** p < 0.001

1. The estimated results of the revised three-factor model are shown in Supplementary Table 9. The f1 factor includes items related to salary increase, daily reimbursement, housing subsidies, and employee entry, and is named "Income Incentives." The f2 factor includes items related to children's education, supplementary housing fund, title appointment, paid leave, recreational activities, and feedback channels, and is named "Job Title Promotion." The f3 factor includes items related to training and further education, and educational advancement, and is named "Education & Training."

Supplementary Table 9 Estimation results of the revised three-factor CFA model (standardized)

| Factors | Items | | Estimate (S.E.) |
| --- | --- | --- | --- |
| f1- Income Incentives | | 1. Raise pay | 0.735***(0.013) |
|  |  | 2. Routine reimbursement | 0.717***(0.014) |
|  |  | 3. Housing subsidy | 0.801***(0.012) |
|  |  | 4. Staff recruitment | 0.508***(0.019) |
| f2- Job Title Promotion | | 5. Children education | 0.550***(0.017) |
|  |  | 6. Supplementary provident fund | 0.686***(0.013) |
|  |  | 9. Appointment of professional title | 0.681***(0.013) |
|  |  | 10. Paid leave | 0.812***(0.011) |
|  |  | 11.Recreational activities | 0.702***(0.013) |
|  |  | 12.Feedback channel | 0.690***(0.013) |
| f3- Education & Training | | 7. Training and further study | 0.892***(0.011) |
|  |  | 8. Academic promotion | 0.792***(0.012) |

1. After completing the model validation and corrections, a high-order fitting of the model was conducted. The results of the high-order factor model fitting are presented in Supplementary Table 10. According to the simulation results, χ2 is 614.18 (P<0.001), and the fitting indices TLI (0.929), AIC (53427.15), BIC (53667.86), CFI (0.949), SRMR (0.040), RMSEA (0.072), etc., basically meet the standard values. There is no significant meaning in establishing a high-order factor model between the appeal factors of medical staff and the attractiveness factors of the middle-aged and young population. The identification of appeal and demand factors is sufficient to support the next stage of modeling work.

Supplementary Table 10 Higher order factor model fitting results

| Model | χ^2^ | *df* | TLI | CFI | AIC | BIC | SRMR | RMSEA (90% CI) |
| --- | --- | --- | --- | --- | --- | --- | --- | --- |
| Two-order model | 614.18*** | 47 | 0.929 | 0.949 | 53427.15 | 53667.86 | 0.040 | 0.078 (0.072, 0.083) |

Note: *** *p* < 0.001

Appendix 4

Supplementary Table 11 Attractiveness and driving force structure

| Side | Factor | Content |
| --- | --- | --- |
| Supply-side (drive force) | Health Care Service (HCS) | Pharmacy services (long prescription, Extended prescription, Drug dispensing service/drug delivery services) |
|  |  | Specialist doctor referral service (Upward referral, Downward referral, Specialist consultation) |
|  |  | Build health records (Establishing health records, Health examination) |
|  |  | Self-health management based on smart devices (Guidance on physical examination checklist consultation, Body mass index assessment and fitness guidance, Open use of smart health devices, Accessibility and visibility of personal digital health records) |
|  | Work-related Diseases Prevention  (WDP) | Eye disease prevention |
|  |  | Cervical spondylosis/frozen shoulder/mouse hand prevention |
|  |  | Breast/cervical disease prevention |
|  |  | Seasonal cold and headache prevention |
|  |  | Chronic gastritis prevention |
|  |  | Sports injury prevention |
|  |  | Traditional Chinese Medicine constitution identification |
|  |  | Tui Na/moxibustion/Cupping/Gua Sha/acupuncture |
|  | Health Education &Consultation  (HEC) | Health information promotion |
|  |  | Group health seminar |
|  |  | Health problem consultation |
|  |  | Psychological problems guidance |
| Demand-side (Attractiveness) | Education & Training (ET) | Training and further education |
|  |  | Academic promotion |
|  | Job Title Promotion (JTP) | Children's education |
|  |  | Supplementary provident fund |
|  |  | Job title appointment |
|  |  | Paid holiday |
|  |  | Recreational activities |
|  |  | Opinion feedback channels |
|  | Income | Salary increase |
|  |  | Daily reimbursement |
|  |  | Housing subsidy |
|  |  | Permanent staff |

Supplementary Table 12 Main parameter settings and source

| Parameter | Unit | Value | Data source |
| --- | --- | --- | --- |
| Number of young and middle-aged people | Person | 16275479 | Shanghai census yearbook 2020 |
| Contract service fee price | RMB | 120 | Policy variable |
| GP allocation ratio | Dmnl | 1500:1 | Model calibration |
| Building service launch time | Year | 2020 | Policy variable |
| Service package price | RMB | 100 | Model calibration |
| Contracted spread rate | Dmnl | 0.08 | Model calibration |
| Personnel adjustment time | Year | 1 | Model calibration |
| Growth rate | Dmnl | 0.1 | Model calibration |
| Minimum drive | Dmnl | 0.5 | Model calibration |
| Standard workload | Dmnl | 0.05 | Model calibration |
| Impact of division of labor coordination | Dmnl | Look up ((0,0.8), (1,2.2)) | Model calibration |
| Income distribution ratio | Dmnl | (0,1) | Policy variable |
